# Influence of *Chlamydia suis* on reproductive outcome in farms with and without tetracycline treatment of sows

**DOI:** 10.1186/s13567-026-01831-w

**Published:** 2026-08-31

**Authors:** Gertrude Baumgartner, René Brunthaler, Lara Ergin, Michaela Koch, Manolis Lyrakis, Karoline Waldner, Andrea Buzanich-Ladinig, Christine Unterweger

**Affiliations:** 1 Clinical Department for Farm Animals and Food System Transformation, Clinical Centre for Population Medicine in Fish, Pig and Poultry, University of Veterinary Medicine, Vienna, Vienna, Austria; 2 Department of Biological Sciences and Pathobiology, Centre for Pathobiology, University of Veterinary Medicine, Vienna, Vienna, Austria; 3 Department of Biological Sciences and Pathobiology, Platform for Bioinformatics and Biostatistics, University of Veterinary Medicine, Vienna, Vienna, Austria; 4 Institute for Veterinary Disease Control, Austrian Agency for Health and Food Safety (AGES), Robert Koch-Gasse 17, 2340 Mödling, Austria

**Keywords:** Genital chlamydiosis, *Chlamydia suis*, *Chlamydia pecorum*, reproductive disorder, tetracycline treatment, tetracycline resistance, *tetA(C)* gene, parity, *Chlamydiaceae*-specific antibodies, genital tract

## Abstract

Porcine genital chlamydiosis is considered a potential cause of reproductive failure, with *Chlamydia (C.) suis* being the most prevalent chlamydial species. To assess its pathogenic relevance, 50 Austrian piglet-producing farms were investigated: 40 farms with reproductive disorders (20 with tetracycline-treatment (tet), 20 without (no-tet)) and 10 clinically unaffected control farms. Samples from 770 sows (tet: *n* = 333, no-tet: *n* = 337, control: *n* =100) including blood, cervical and rectal swabs, dust samples and one pooled nursery fecal sample per farm were analyzed. Thirty-seven reproductive-tracts and six abortion cases were examined. Species-specific real-time PCR detected cervical *C. suis* shedding in 2.1% of sows (tet: *n* =5, no-tet: *n* = 10, control: *n* = 1). Rectal shedding was found on 45 farms and in 39.7% of sows overall (tet: *n* = 37.8%, no-tet: *n* = 39.2%, control: *n* = 48%). *C. pecorum* DNA was identified in one cervical and seven rectal swabs. *Chlamydiaceae*-specific antibodies were detected by complement fixation test on all farms and in 49.8% of sows overall, without association between study group and shedding or serological status. *C. suis* DNA was detected in 28% of dust and 96% of nursery fecal samples, whereas reproductive tract and abortion material were largely PCR-negative. Analyzing 42 *C. suis* isolates high rates of tetracycline resistance were found in all study groups via *tetA(C)* PCR (tet: *n* = 93.75%, no-tet: *n* = 78.3%, control: *n* = 100%) and in vitro susceptibility testing (tet: *n* = 75%, no-tet: *n* = 60.8%, control: *n* = 100%). Parity had negative effects on clinical signs, rectal shedding and antibody expression. *C. suis* appears to play a limited role in reproductive failure, while routine tetracycline treatment should be critically reconsidered due to frequent resistance.

**Supplementary Information:**

The online version contains supplementary material available at https://doi.org/10.1186/s13567-026-01831-w.

## Introduction

*Chlamydia (C.)* spp. are obligate intracellular, Gram-negative bacteria causing a wide spectrum of diseases in humans and animals [1]. In pigs, several species have been described, including *C. suis*, *C. abortus*, *C. pecorum*, and *C. psittaci*, with *C. suis* representing by far the most prevalent and epidemiologically relevant species in swine populations [1–3]. Chlamydial infections in pigs have been associated with a variety of clinical manifestations affecting multiple organ systems, including conjunctivitis [4], pneumonia [5], and enteritis [6]. The gastrointestinal system of pigs serves as a reservoir for *C. suis* [2, 7], with rectal shedding peaking in nursery piglets and finishers [8]. The reproductive tract represents the primary target of *C. suis,* with infections linked to substantial reproductive losses, as reported in studies [9–15] and by practicing swine veterinarians [16]. In female pigs, genital chlamydiosis is associated with abortions [14, 17], mummification [1], perinatal mortality [18], endometritis, vaginal discharge [1], increased return-to-estrus rates [9, 10], and overall poor reproductive performance [12]. Chlamydiae have been detected in aborted fetal material [14], and chlamydia DNA has also been identified in vaginal swabs [19] and in the uteri and oviducts of slaughtered sows [10]. A molecular and immunohistochemical study has further demonstrated the presence of chlamydial DNA and antigen in different layers of the uterine wall [10], and an experimental in vitro study has confirmed the susceptibility of porcine uterine epithelial cells to *C. suis* infection [20]. Chlamydiae have also been detected in the porcine oviduct [10], suggesting that, analogous to humans and other animal species, the oviduct may serve as an additional target organ. Despite this growing body of evidence, the causal role of *C. suis* in the pathogenesis of reproductive failure in sows remains incompletely understood, and causal links between infection, tissue pathology, and impaired fertility are still difficult to establish [11]. It can generally be assumed that the prevalence of *C. suis* in pig farms is very high, irrespective of the production system [2, 19]. *C. suis* is also frequently found in environmental samples, such as dust, although environmental viability appears limited [21]. Given the consistent rectal shedding of *C. suis* [8], fecal–oral transmission is considered a likely route of spread, with bacteria potentially ascending from the vagina into the uterus and upper reproductive tract during periods of cervical opening, similarly described in *C. trachomatis* cases in women [22].

Diagnosis of *C. suis* induced reproductive failure remains challenging. PCR detects chlamydial DNA, but cannot confirm viability, and optimal sampling strategies are poorly defined. Serology is limited by lack of validated assays, and positive results may reflect persistent intestinal colonization rather than active genital infection. Histopathology and immunohistochemistry provide more definitive evidence of infection and tissue involvement [10].

Treatment relies on antibiotics with intracellular activity, primarily tetracyclines and macrolides. Tetracyclines have been used in livestock since the 1950 s, and although the *C. suis* type strain S45, isolated from an Austrian pig in the 1960 s, is sensitive for tetracycline [23], *C. suis* strains expressing the *tetA(C)* resistance gene are increasingly reported [24, 25]*.* The *tetA(C)* gene encodes a TET efflux pump, and has been horizontally transferred to other *Chlamydiaceae* species in vitro [24, 26], posing a possible One Health concern. This is of particular relevance in pig production, where oxytetracycline or doxycycline are commonly administered via feed or water to sows with fertility problems during the insemination period [27]. In this context, the increasing occurrence of tetracycline resistance may compromise treatment efficacy. Furthermore, the absence of controlled infection studies in fertile sows precludes evidence-based guidance on treatment duration and dosage, rendering antimicrobial efficacy unpredictable.

This study aimed to provide the first comprehensive assessment of *C. suis* in Austrian pig farms with suspected genital chlamydiosis or general fertility problems, with particular emphasis on comparing findings between farms with and without routine tetracycline use. PCR and serological detection rates were determined, viable strains were isolated, and tetracycline resistance was characterized. Comparisons were performed at both herd and individual level, including farms with and without reproductive problems, farms with and without tetracycline use, and sows with and without reproductive disorders within the same herd.

Collectively, these data should provide a detailed baseline on *C. suis* epidemiology in pigs, and antimicrobial resistance patterns. Given the global ubiquity of *C. suis*, data generated in Austria can be contextualized internationally, as detection rates and resistance patterns are likely comparable across other regions.

## Materials and methods

The animal study protocol was approved by the Ethics and Animal Welfare Committee of the University of Veterinary Medicine, Vienna in accordance with the University`s guidelines for Good Scientific Practice and authorized by the Austrian Federal Ministry of Education, Science and Research (ref BMBWF 2023–0.623.493) in accordance with current legislature. All participants were informed about aim and structure of the study and gave their consent to conduct the study on their farms and to publish the acquired data.

### Study population

In total, 770 sows from 50 Austrian pig farms were included in this study. Sampling was conducted between September 2023 and June 2024. All farms were located in the three main pig-producing federal states of Austria, namely Upper Austria (*n* = 17), Lower Austria (*n* = 18), and Styria (*n* = 15). Of the 50 farms investigated, 40 farms exhibited reproductive problems, while 10 farms served as controls without reproductive disorders. On all farms with reproductive problems, the attending herd veterinarian had suspected a potential involvement of *C. suis* as a contributing pathogen. Reproductive problems were defined according to previous reports [28] as farrowing rate below 85%, return-to-estrus rate exceeding 10%, and/or abortion rate above 10%. Forty farms with reproductive problems were further stratified based on their tetracycline usage. Study group “tet” comprised 20 farms in which sows had received tetracycline treatment within the preceding six months. Study group “no-tet” included 20 farms with reproductive problems but no tetracycline treatment of sows during the past two years. The control group (“control”) consisted of 10 farms without reproductive problems and without tetracycline administration to sows within the last two years. Further characteristics of study farms have been described elsewhere [27].

### On farm sample collection

To assess the environmental chlamydial load, one dust sample was collected from the surfaces within the breeding unit on each farm. Additionally, one pooled fecal sample was collected from each nursery unit and used as positive control. On farms with active reproductive disorders, up to 20 sows per farm were sampled in the insemination unit. The number of sows sampled per farm was adjusted according to the number of clinically affected animals, with sows showing reproductive disorders comprising 50% of the sampled population. Whenever possible, ten sows with reproductive problems and ten sows without reproductive problems were included per farm. In control farms, ten sows from the insemination unit were randomly selected for sampling. From each sow, a blood sample was collected from the jugular vein, and flocked swabs (FLOQSwabs Minitip Flocked Swab 501CS0, Copan Italia S.p.A., 88 Brescia, Italy) were taken from the cervix and rectum. Cervical sampling was performed using reusable plastic specula, which were disinfected between animals by immersion in a Vircon®-S solution for five minutes. On-farm, all swabs were placed in sucrose-phosphate glutamate (SPG) transport medium as previously described [2] in sterile Eppendorf® tubes and stored at −80 °C on the day of collection until further processing. Serum samples were stored at −20 °C.

### Additional samples

Aborted litters of both the tet (*n* = 2) and no-tet (*n* = 4) study groups were submitted for C*hlamydia* spp. screening by PCR. From each aborted litter, pooled tissue samples comprising fetal liver, lung and kidney as well as placental tissue were collected and stored at − 20 °C until DNA extraction. In addition, uteri from slaughtered sows (*n* = 37) with a history of reproductive disorders were submitted for further examination. Standardized tissue samples were collected from each uterus for PCR analysis and formalin fixation for histopathological examination. Tissue sampling was performed according to a slightly modified protocol based on Kauffold et al. [10]. Detailed information on sampling sites and procedures is provided in Table 1.

**Table 1:** **Sampling sites and methods for uterine tissue collection for PCR and histopathology**

Method	Oviduct left	Oviduct right	Uterine horn left	Uterine horn right	Cervix
PCR	flocked swab	flocked swab	mucosal scraping of whole horn	mucosal scraping of whole horn	flocked swab
Formalin fixation	1 cm of isthmus	1 cm of isthmus	•1 cm from middle of proximal half •1 cm from middle of distal half	•1 cm from middle of proximal half •1 cm from middle of distal half	−

Tissue samples were fixed in 10% neutral-buffered formalin for histological examination. After adequate fixation, the samples were routinely processed and embedded in paraffin wax. Sections were cut at approximately 1–2 µm thickness and stained using standard hematoxylin–eosin (H&E). All stained sections were evaluated in a blinded manner by a veterinary pathologist.

### DNA extraction and PCR

Before withdrawing 100 µL of sample material for extraction, Eppendorf® tubes containing swab, sample material medium and three solid glass beads as described above, were vortexed and briefly centrifuged. DNA was extracted from all cervical swabs (*n* = 770), rectal swabs (*n* = 770), dust (*n* = 50), and fecal samples (*n* = 50) using the QIAamp DNA Mini Kit (Qiagen, Hilden, Germany) according to the manufacturer’s instructions with a final elution volume of 100 µL. Following extraction, all samples were screened using a *Chlamydiaceae* family-specific real-time PCR targeting the 23S rRNA gene and including an internal amplification control, as previously described [29]. A sixfold serial dilution of *C. abortus* genomic DNA, was used to generate the standard curve [2]. Previously characterized positive reference material served as the positive control, and DNA-free water was included as a negative control. The threshold line was set at 0.1 for all runs, and *Chlamydiaceae* copy numbers per mL were calculated for each sample as previously described [30]. Total number of cycles was 45, all samples with a cycle threshold (Ct) value < 45 were subsequently analyzed using a *C. suis*-specific 23S rRNA real-time and a *C. pecorum*-specific ompA real-time PCR [31]. DNA yield was expressed as genome equivalents (GE)/mL.

### Chlamydia isolation in cell culture

Genital samples with *Chlamydiaceae* Ct-values < 35 were subjected to isolation in cell culture. Additionally, at least two rectal swabs per farm with Ct-values < 35, and every nursery fecal pool sample and dust sample with Ct-value < 35 was subjected to isolation.

Briefly, samples in the same Eppendorf® tubes used for extraction were again vortexed and briefly centrifuged, after which 200 µL of sample material was withdrawn. Isolation was performed using the LLC-MK2 continuous rhesus monkey kidney epithelial cell line (kindly provided by IZSLER, Brescia, Italy) using standard techniques [32] modified by [25]. Samples were considered negative for viable *Chlamydia* spp. when no inclusions were observed after three serial passages and qPCR results remained negative. All samples were stored at −80 °C in at least duplicate aliquots for downstream analyses and qPCR-based confirmation of the presence or absence of chlamydial DNA.

### Tetracycline susceptibility testing

#### TetA (C) gene PCR

Conventional *tetA(C)* PCR was performed to detect the tetracycline resistance gene *tetA(C)* in chlamydial isolates previously propagated in LLC-MK2 cell cultures. Genomic DNA was extracted using the QIAamp DNA Mini Kit (Qiagen, Hilden, Germany) according to the manufacturer’s instructions. PCR reactions were prepared in a final volume of 10 µL, containing 1.5 µL of template DNA, 10 µM of each primer (CS43: AGCACTGTCCGACCGCTTTG; CS47: TCCTCGCCGAAAATGACCC), as originally described by Dugan et al. [24] 1× AmpliTaq Gold 360 Master Mix (Thermo Fisher Scientific, Waltham, MA, USA), and nuclease-free water. Amplification was conducted in a T3000 thermocycler (Biometra, Göttingen, Germany) with an initial denaturation at 95 °C for 5 min, followed by 35 cycles of denaturation at 95 °C for 30 s, annealing at 59 °C for 30 s, and extension at 72 °C for 1 min, and a final extension at 72 °C for 10 min. PCR products were separated on a 1.5% agarose gel in 1× TAE buffer containing GelRed® nucleic acid stain and visualized using a Gel iX Imager system (INTAS Science Imaging Instruments GmbH, Göttingen, Germany). Amplicon sizes were determined using a 1 kb DNA ladder (GeneRuler™, Thermo Fisher Scientific).

### Tetracycline susceptibility screening in vitro

For in vitro tetracycline susceptibility testing, inclusion-forming units per milliliter (IFU/mL) were determined for each isolate in advance to standardize the multiplicity of infection (MOI) across all experiments and to ensure comparability between isolates. The experimental setup and resistance testing were performed according to previously published “tet screen” protocols with minor modifications [25, 33, 34]. Tetracycline hydrochloride powder (Sigma-Aldrich, St. Louis, MO, USA) was dissolved in dimethyl sulfoxide (DMSO, Sigma-Aldrich) to obtain a final concentration of 10 mg/mL, filter-sterilized using a 0.22 µm syringe filter, aliquoted, and stored at −20 °C. Tetracycline susceptibility testing was performed in 96-well plates, with each isolate tested in duplicate. Briefly, LLC-MK2 cells were seeded into 96-well plates 24 h prior to infection, and confluent monolayers were subsequently infected at an MOI of approximately 0.5. Following centrifugation (1 h, 1000* g*, 25 °C), the inoculum was removed and replaced with cycloheximide-containing chlamydial infection medium with or without tetracycline at final concentrations of 0.125 or 0.5 µg/mL. For each isolate, mock-infected wells containing infection medium only served as controls. Cultures were incubated at 37 °C in 5% CO_2_ for 30–34 h, fixed in pre-chilled methanol (−20 °C) for 10 min, and further processed for immunofluorescence staining as previously described [33].

Widefield fluorescence imaging was performed using an inverted Ti2-E microscope (Nikon Europe, Amstelveen, Netherlands) equipped with a DS-Qi2 CMOS camera. Images were acquired using a 10X Plan Apochromat objective (NA = 0.45). Fluorescence excitation was provided by a SPECTRA III LED light engine (Lumencor, Beaverton, OR, USA) using 390 nm and 475 nm excitation lines. Emission signals were collected using the following filter sets: DAPI (432/18 nm) and Alexa Fluor 488 (515/15 nm). Image acquisition was performed under identical exposure settings for all samples within each experimental condition. Images of entire wells in 96-well plates were automatically tiled and stitched driven by the Nikon NIS elements software module JOBS.

Stitched images were analyzed using the software arivis Pro (Carl Zeiss Microscopy GmbH, Vienna, Austria). Nuclei were detected using the Cellpose Based Segmenter operation. Green fluorescent signals were segmented by applying an intensity threshold. Segmented objects were subsequently classified using the Machine Learning Object Classifier tool based on size and shape parameters.

Inclusion counts were quantified and compared between tetracycline-exposed and untreated control cultures. Isolates were classified as “tetracycline susceptible” if inclusion numbers were reduced by ≥ 90% following tetracycline exposure, “intermediately susceptible” if inclusion reduction ranged between 50% and < 90%, and “tetracycline-resistant” if inclusion numbers decreased by < 50% compared with the corresponding untreated control cultures, according to previously established criteria [33, 35].

### Complement fixation test (CFT)

Serum samples were stored at −20 °C until analysis for antibodies against *Chlamydiaceae* using an in-house complement fixation test at the Institute for Veterinary Disease Control of the Austrian Agency for Health and Food Safety GmbH (AGES). Prior to testing, serum samples were diluted 1:5 in CFT buffer (Innovative Diagnostics, France) and heat-inactivated in a water bath at 60 °C for 30 min. After equilibration to room temperature, a two-fold serial dilution ranging from 1:5 up to 1:160 was prepared for each sample in microtiter plates. For each dilution step 25 µL of complement and *C. suis* antigen were added at predetermined concentrations to 25 µL of diluted sample. Anti-complementary activity was assessed in separate wells for each sample in the dilutions 1:5 and 1:10 by adding CFT buffer instead of antigen. A positive and negative control as well as antigen and complement control wells were included on each test plate. Following incubation for 16–20 h at 2–8 °C, 50 µL of 2% sensitized sheep red blood cells were added to each well, and plates were incubated at 37 °C for 30 min. Plates were then centrifuged for 5 min at 450 × *g* and examined using a reading mirror. Titers were recorded as the highest dilution showing inhibition of hemolysis.

### Statistics and data processing

All data were first put into a spreadsheet program (Microsoft Office^©^ Excel 365), all graphs were created in GraphPad Prism (version 10.6.1) or Excel (Microsoft Office^©^ 365).

Statistical analysis was performed in R (R version 4.5.2) [36]. Significance was declared at 5% cut off. Identification of *C. suis* rectal shedding (in 710 sows) and expression of *Chlamydiaceae*-specific antibodies (in 668 sows) were evaluated via separate logistic regression mixed models (package *lme4*, version 1.1.37, function *glmer*, option family = binomial (link = “logit”), control = glmerControl(optimizer = "bobyqa", optCtrl = list(maxfun = 10,000))) [37]. Both variables were fitted as binary responses. Group (3 groups) and parity were fitted as fixed effects. Farm ID (46 farms since parity data were not available for all farms) was fitted as random intercept to account for the covariance structure in the data, as there are multiple observations per farm. Furthermore, parity was fitted as random slope within farm ID.

Identification of fertility issues (in 668 sows) was also evaluated via a logistic regression mixed model. Fertility issues were fitted as a binary response. The identification of *C. suis* cervical and rectal shedding, expression of *Chlamydiaceae*-specific antibodies and parity were fitted as fixed effects. Farm ID (46 farms since parity data were not available for all farms) was fitted as random intercept to account for the covariance structure in the data, as there are multiple observations per farm. Furthermore, dummy-coded centered categorical fixed effects and parity were fitted as random slopes within farm ID.

Assumptions about the random intercepts and random slopes were visually evaluated. Multicollinearity between the fixed effects was evaluated via variance-inflation factors (package *car*, version 3.1.3, function *vif*) [38]. Stability of each model was evaluated with a leave-one-out strategy by comparing fitted values based on the model using all data to those of models based on data with farm IDs (random intercept) excluded one at a time. Overall group significance was evaluated via a likelihood ratio test using the function *anova*, comparing the full model to a reduced model without the group as fixed effect. Pairwise comparisons between the levels of categorical fixed effects for each model were conducted via the estimated marginal means (package *emmeans*, version 1.11.0, function *emmeans*) [39]. Pairwise comparisons between groups were corrected for multiple testing via the Tukey method.

Cervical shedding of *C. suis* was not evaluated due to low identification. The association between the identification of *C. suis* rectal shedding and expression of *Chlamydiaceae*-specific antibodies (in 727 sows from 50 farms) was evaluated via the Cochran-Mantel–Haenszel chi-squared test (function *mantelhaen.test*) that took Farm ID into account.

## Results

### Clinical samples

A total of 333 sows (163 clinically affected and 170 unaffected) were sampled on 20 farms with routine tetracycline treatment for reproductive failure (tet). On 20 farms without tetracycline treatment but with reproductive failure (no-tet), 337 sows were sampled (164 clinically affected, 173 unaffected). In the control group (10 farms without a history of reproductive failure), 100 sows were sampled, all of which were clinically unaffected. A detailed overview of PCR results of clinical samples can be found in Additional file 1.

### Cervical *C. suis *and* C. pecorum* shedding

Cervical *C. suis* DNA shedding was found in 2.08% of sows (16 sows of ten different farms). Five positive sows originated from tet farms, ten from no-tet farms and one from a control farm. *C. pecorum* DNA was detected in one cervical swab obtained from a sow exhibiting repeat breeding. Parity among positive sows ranged from 0 to 8. Eight of the positive animals showed no clinical signs of impaired fertility. Among the remaining sows, six were rebreeders and two had recently aborted (Table 2).

**Table 2:** **Sample characteristics of sows with cervical Chlamydia shedding**

Sow ID	Farm ID	Study group	Parity	Clinical signs	Cervix				Rectum			DNA yield (GE/mL)	Titer CFT
					*C. suis*		*C. pecorum*		*C. suis*		*C. pecorum*		
					Ct-value	DNA yield (GE/mL)	Ct-value	DNA yield (GE/mL)	Ct-value	DNA yield (GE/mL)	Ct-value		
201*	4	tet	2	Rebreeding	31.69	231.92	−	−	38.05	3.26	−	−	1:10
218	4	tet	0	None	40.41	0.57	−	−	28.34	2,755.67	−	−	1:5
466	9	tet	6	Abortion	39.95	0.79	−	−	−	−	−	−	AC
531	12	tet	0	Rebreeding	−	−	37	15.44	−	−	29.99	165	n.a
548	12	tet	0	Rebreeding	39.99	0.76	−	−	28.69	2,652.46	−	−	1:10
751	18	tet	8	Rebreeding	33.46	68.8	−	−	−	−	−	−	1:5
42	22	no-tet	6	None	38.31	2.44	−	−	−	−	−	−	neg
48	22	no-tet	1	Abortion	34.70	29.23	−	−	29.53	787.42	−	−	neg
94	24	no-tet	2	Rebreeding	39.85	0.84	−	−	−	−	−	−	1:10
95	24	no-tet	1	Rebreeding	27.31	4,765.60	−	−	−	−	−	−	1:10
105	24	no-tet	5	None	40.23	0.65	−	−	34.77	26.92	−	−	neg
107	24	no-tet	2	None	31.04	363.24	−	−	29.81	657.78	−	−	1:5
109	24	no-tet	1	None	39.03	1.48	−	−	31.18	272.37	−	−	AC
283	29	no-tet	4	None	34.53	32.93	−	−	−	−	−	−	neg
332	31	no-tet	3	None	38.43	2.24	−	−	30.36	741.35	−	−	AC
603	38	no-tet	1	Rebreeding	39.96	0.78	−	−	31.44	555.88	31.84	31	1:5
855	48	control	7	None	38.91	1.6	−	−	−	−	−	−	neg

^*^: *C. suis* was successfully isolated in cell culture from the cervical swab, *AC* Anti-complementary activity, *n.a*. not analyzed −: Ct-value > 45/DNA yield not detectable

GE genome equivalents, CFT complement fixation test

### Rectal *C. suis* and *C. pecorum* shedding of sows

**Figure 1:**
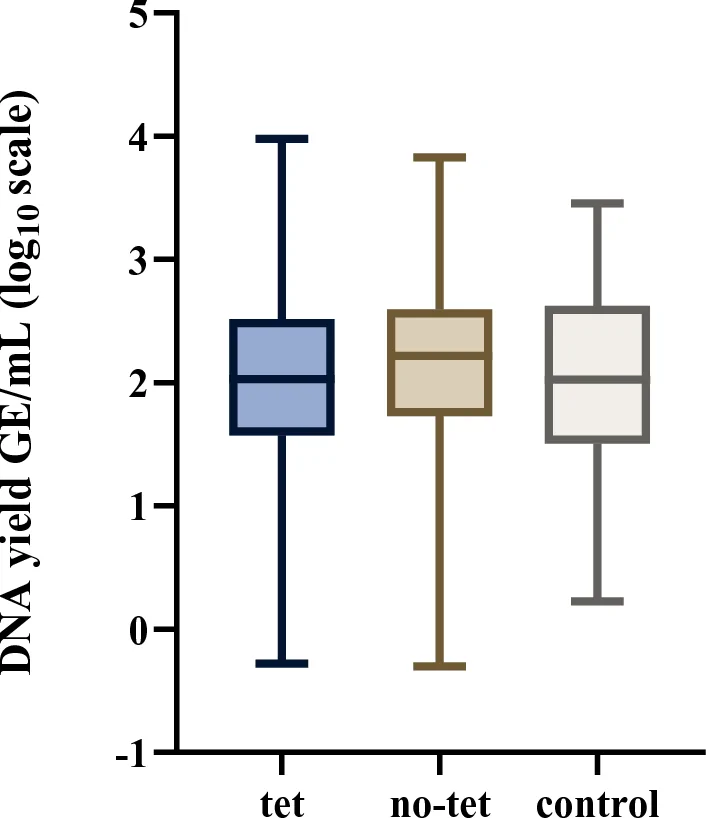
**Log**_**10**_**-scaled *C. suis* DNA yield (Genome equivalents (GE)mL) of positive rectal swabs from sows of different study groups (tet, no-tet, control). Rectal shedding of *C. pecorum* was identified in seven sows from four farms across all study groups, detailed information is provided in Table** 3**.**

Rectal shedding of *C. suis* DNA was observed on 45 farms across all study groups (Figure 1) and in 39.7% of all tested sows. On farms with regular tetracycline use (tet), 37.8% of sows (126/333) tested positive for rectal *C. suis* DNA, compared with 39.2% (132/337) of sows on farms without tetracycline use (no-tet). In control farms, 48% of sows (48/100) shed rectally (Figure 1). No association was identified between study group and rectal shedding of *C. suis* (*p* = 0.769).

**Table 3:** **Microbiological and clinical findings in sows with rectal shedding of* C. pecorum***

Sow ID	Farm ID	Study group	Parity	Clinical signs	Cervix				Rectum				Titer CFT
					*C. suis*		*C. pecorum*		*C. suis*		*C. pecorum*		
					Ct-value	DNA yield (GE/mL)	Ct-value	DNA yield (GE/mL)	Ct-value	DNA yield (GE/mL)	Ct-value	DNA yield (GE/mL)	
531	12	tet	0	rebreeding	-	-	36.90	15.44	-	-	29.99	117.40	n.a
534	12	tet	0	none	-	-	-	-	30.74	825.25	40.00	0.06	1:5
115	25	no-tet	1	rebreeding	-	-	-	-	30.61	394.58	32.83	63.38	1:20
116	25	no-tet	1	rebreeding	-	-	-	-	29.78	670.19	39.67	0.53	1:10
128	25	no-tet	9	rebreeding	-	-	-	-	38.93	1.85	41.52	0.15	1:10
603	38	no-tet	1	rebreeding	39.96	0.78	-	-	31.44	555.88	31.84	31.00	1:5
676	44	control	0	none	-	-	-	-	31.16	424.71	29.75	164.61	1:10

−: Ct-value > 45/DNA yield not measurable

CFT complement fixation test, GE genome equivalents

### Expression of *Chlamydiaceae*-specific antibodies

**Figure 2:**
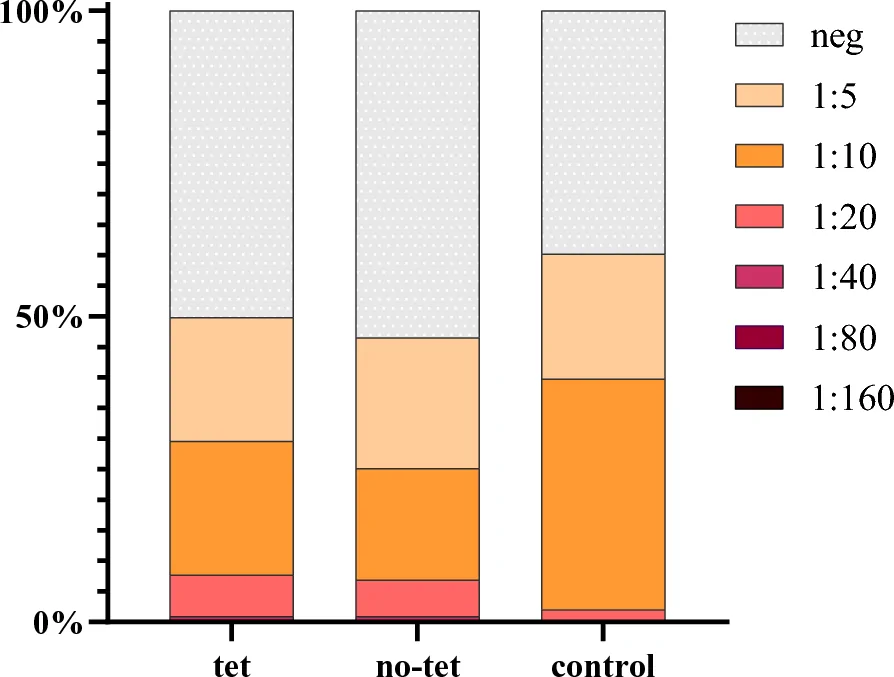
**Percentage of *Chlamydiaceae***-**specific antibody titer distribution among the three study groups (tet, no-tet, control).**

Using CFT, antibodies against *Chlamydiaceae* were detected in sows from all study groups and across all farms (Figure 2 and Table 4). A total of 727 blood samples were analyzed; 43 samples were excluded due to autoagglutination or insufficient quality. Overall, 49.8% (362/727) of sows had detectable antibody titers. Seropositivity was observed in 49.8% (155/311) of sows in the tet group, 46.5% (148/318) of the no-tet group, and 60.2% (59/98) in the control group. No association was observed between study groups and expression of *Chlamydiaceae*-specific antibodies (*p* = 0.226).

**Table 4:** **Detailed overview of** ***Chlamydiaceae***-**specific antibody titer distributions of all analyzed blood samples (*n *= 727) among 3 study groups (tet, no-tet, control)**

CFT titer	tet		no-tet		control	
	no. of sows	(%)	no. of sows	(%)	no. of sows	(%)
Neg	156	(50.2)	170	(53.5)	39	(39.8)
1:5	63	(20.3)	68	(21.4)	20	(20.4)
1:10	68	(21.9)	58	(18.2)	37	(37.8)
1:20	21	(6.8)	19	(6.0)	2	(2.0)
1:40	2	(0.6)	3	(0.9)	0	(0.0)
1:80	0	(0.0)	0	(0.0)	0	(0.0)
1:160	1	(0.3)	0	(0.0)	0	(0.0)

CFT complement fixation test, neg negative

**Figure 3:**
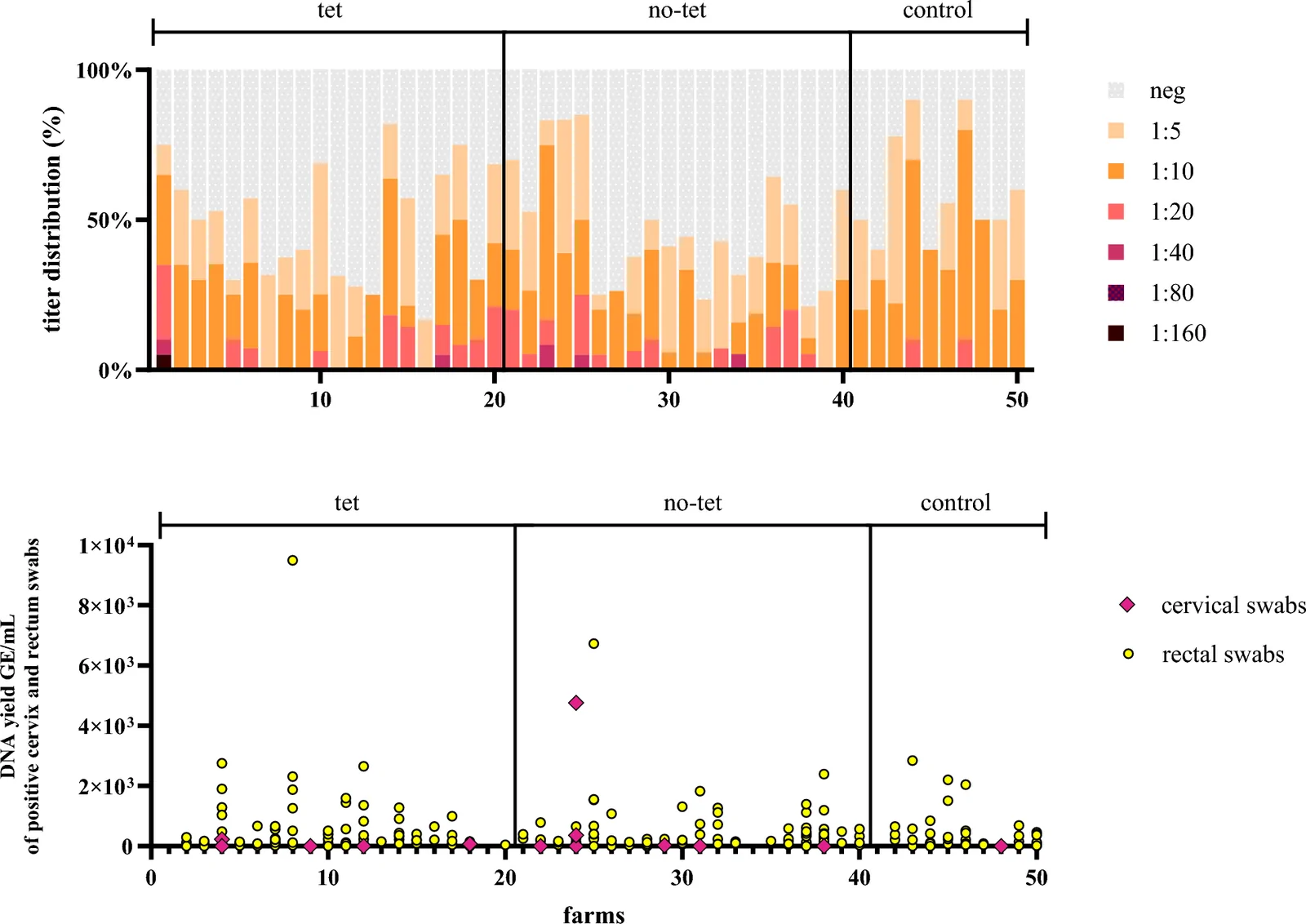
***Chlamydiaceae***-**specific antibody titers and *C. suis* positive cervical and rectal swabs found per farm.** **A** *Chlamydiaceae*-specific antibody titer distribution per farm, determined by complement fixation test; **B** *C. suis* DNA yield (GE/mL) from rectal and cervical swabs per farm.

At least two sows with *Chlamydiaceae*-specific antibodies were detected on every farm, whereas sows with rectal shedding of *C. suis* were not present on all farms. A detailed comparison across farms is shown in Figure 3A, B. No association was observed between antibody presence and rectal shedding (odds ratio = 1.017, *p* = 1).

### Fecal samples from the nursery and environmental dust from breeding pen

**Figure 4:**
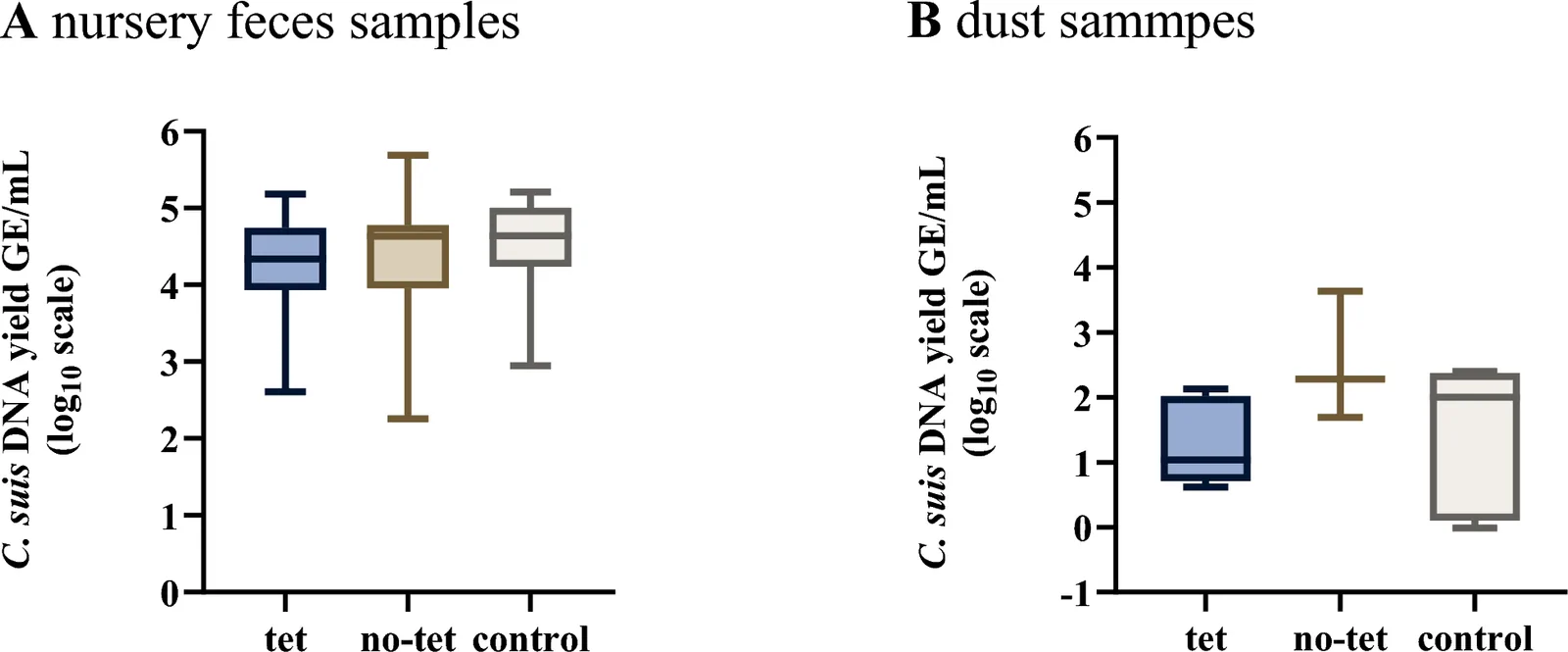
**DNA yield (GE/mL) found in positive nursery feces samples and dust samples.** **A** Log_10_-scaled *C. suis* DNA yield (GE/mL) in positive fecal samples from nursery piglets across study groups. **B** Log_10_-scaled *C. suis* DNA yield (GE/mL) in positive dust samples collected from breeding rooms of the study groups.

Pooled fecal samples from nursery piglets tested positive for *C. suis* on 48 farms (96%). Specifically, 19 of 20 samples (95%) from tet farms and 19 of 20 samples (95%) from no-tet farms tested positive, while all ten samples (100%) from control farms tested positive. *C. pecorum* was detected in a single sample from a tet farm (Figure 4A).

Of 50 dust samples analyzed, 14 (28%) tested positive for *C. suis*. Among these, five of 20 samples (25%) from tet farms, 3 of 20 samples (15%) from the no-tet farms, and 6 of 10 samples (60%) from control farms tested positive (Figure 4B). Co-occurrence with *C. pecorum* was identified in two *C. suis* positive dust samples from no-tet and control farms. A detailed overview of PCR results of fecal and dust samples can be found in Additional file 2.

### Clinical signs and parity

Detected genital (*p* = 0.416) or rectal (*p* = 0.379) *C. suis* shedding had no effect on clinical signs. Expression of antibodies also had no effect on clinical signs (*p* = 0.646). At the time of sampling 25 sows (tet: 12; no-tet: 13) from 14 farms exhibited purulent vaginal discharge. Among these sows, all cervical swabs tested negative for *C. suis* and *C. pecorum* in qPCR. However, 6/25 sows (24%) shed *C. suis* DNA rectally, but none shed *C. pecorum* DNA rectally. Furthermore, *Chlamydiaceae*-specific antibodies were detected in 9 out of 24 sows, resulting in a seroprevalence of 37.5%.

**Figure 5:**
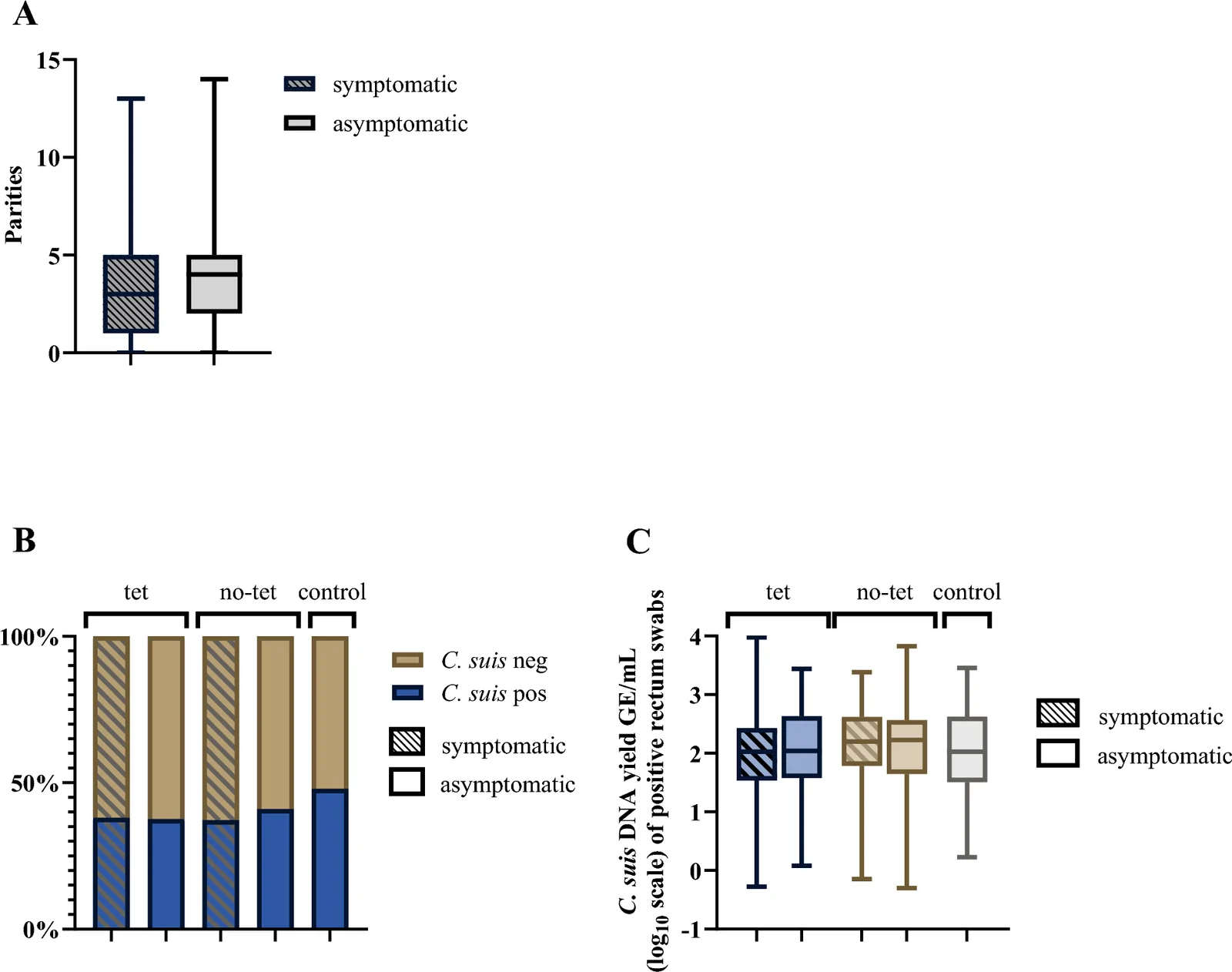
**Characteristics and PCR results found in sows with different clinical status.** **A** Distribution of parity in sows categorized by presence or absence of reproductive disorders. **B** Proportion of symptomatic and asymptomatic sows exhibiting rectal *C. suis* shedding across the different study groups. Dark blue shaded bars represent the percentage of positive samples, while lighter brown shades indicate negative samples; **C** Log_10_-scaled *C. suis* DNA load in rectal swabs of sows, stratified by study group and clinical status.

Parity data were available for 710 sows (range: 0–14). A negative effect of parity on clinical signs was observed (slope = −0.149, *p* = 0.012) (Figure 5A). Furthermore, parity was found to have a negative effect on rectal *C. suis* shedding (slope = −0.132, *p* = 0.0004) as well as a negative effect on presence of *Chlamydiaceae*-specific antibodies (slope = −0.078, *p* = 0.0225). Within the study cohorts, the control group exhibited the highest rate of *C. suis* positive rectal swabs at 48% (Figure 5B), while median DNA loads ranged between 1.69 × 10^2^ and 1.06 × 10^2^ (Figure 5C).

### Isolation in cell culture and antibiotic susceptibility

**Figure 6:**
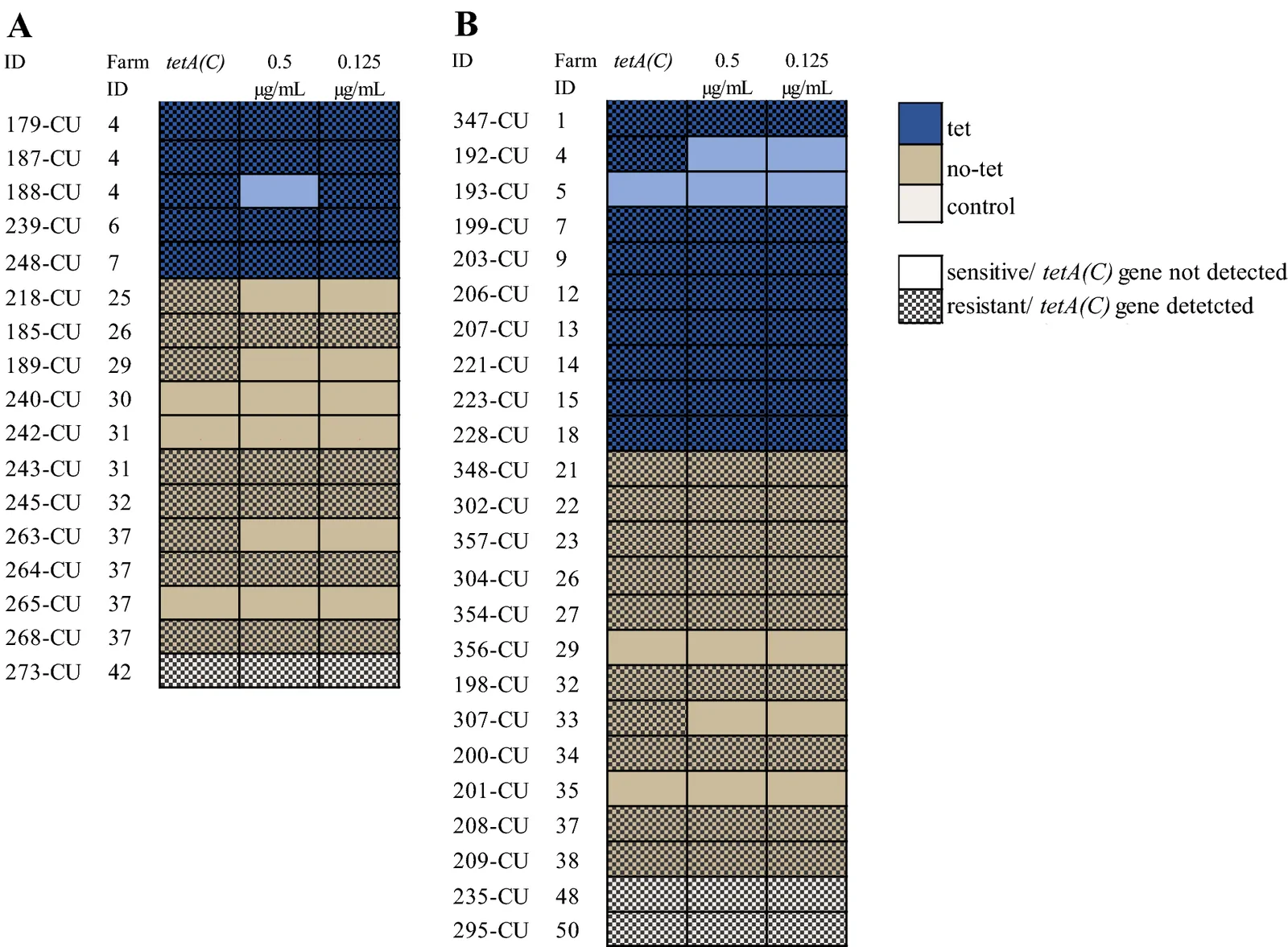
**Tetracycline susceptibility profiles and** ***tetA(C)*** **prevalence in** ***C. suis*** **isolates from rectal swabs or fecal samples.** Isolates were evaluated at two tetracycline concentrations (0.125 µL/mL and 0.5 µL/mL). Blue: samples from tet farms; brown: samples from no-tet farms; white: samples from control farms. Grid squares: indicate phenotypic resistance or the presence of the *tetA(C)* gene; plain square: indicate phenotypic sensitivity or absence of the *tetA(C)* gene. Isolates derived from rectal swabs of sows **A** or from nursery fecal samples **B**.

In total, 49 *Chlamydiaceae* strains were isolated from 49 samples originating from 29 farms (Additional file 3). Specifically, 17 isolates originated from eleven tet farms, 29 from 15 no-tet farms, and three from three control farms. A detailed overview of all 49 isolates is provided in Tables 5–6 and Figure 6. *C. suis* was successfully isolated from a single cervical swab of a rebreeding sow with an antibody titer of 1:10. This sow was from a tet farm. Furthermore, *C. suis* was isolated from six rectal swabs of sows of the tet group, 16 rectal swabs of sows of the no-tet group, and one rectal swab of a sow from the control group. From nursery piglets, *C. suis* was isolated from 25 fecal samples, including 10 isolates from the tet group, 13 from the no-tet group, and 2 from the control group. A total of 42 isolates were identified as *C. suis,* and two isolates were identified as *C. pecorum*. Co-culture of both *C. suis* and *C. pecorum* was achieved from four rectal and one fecal sample (Tables 5 and 6).

**Table 5:** **Characteristics of isolates from cervical (*n* = 1) and rectal (*n* = 23) swabs of sows**

ID	Farm ID	Study group	Parity	Clinical signs	CFT titer	Location of sampling	*C. suis*		*C. pecorum*		Susceptibility to tetracycline		
							Ct- value	DNA yield (GE/mL)	Ct-value	DNA yield (GE/mL)	*tetA(C)* gene	0.5 µg/mL	0.125 µg/mL
177-CU	4	tet	2	rebreeding	1:10	cervix	20.36	2.30E+08	−	−	+	+	+
179-CU	4	tet	0	none	1:5	rectum	16.12	3.99E+09	−	−	+	+	+
187-CU	4	tet	2	none	1:10	rectum	15.01	8.42E+09	−	−	+	+	+
188-CU	4	tet	1	none	neg	rectum	19.72	3.55E+08	−	−	+	−	+
239-CU	6	tet	5	rebreeding	1:5	rectum	22.58	5.18E+07	−	−	+	+	+
248-CU	7	tet	1	rebreeding	neg	rectum	20.2	2.57E+08	−	−	+	+	+
259-CU	12	tet	6	none	neg	rectum	39.14	7.42E+02	18.92	2.75E+08	n.a	n.a	n.a
215-CU	25	no-tet	1	rebreeding	1:20	rectum	−	−	16.65	1.19E+09	n.a	n.a	n.a
217-CU	25	no-tet	3	none	1:20	rectum	16.84	2.46E+09	37.3	1.91E+03	n.a	n.a	n.a
218-CU	25	no-tet	1	none	1:10	rectum	15.08	8.05E+09	−	−	+	−	−
185-CU	26	no-tet	2	rebreeding	neg	rectum	14.46	1.22E+10	−	−	+	+	+
189-CU	29	no-tet	1	none	n.a	rectum	16.04	4.22E+09	−	−	+	−	−
240-CU	30	no-tet	3	none	neg	rectum	19.09	5.43E+08	−	−	−	−	−
242-CU	31	no-tet	8	none	neg	rectum	27.69	1.65E+06	−	−	+	+	+
243-CU	31	no-tet	3	none	n.a	rectum	24.07	1.90E+07	−	−	+	+	+
245-CU	32	no-tet	1	rebreeding	n.a	rectum	26.23	4.42E+06	−	−	+	+	+
263-CU	37	no-tet	1	none	1:5	rectum	17.09	2.08E+09	−	−	+	−	−
264-CU	37	no-tet	0	rebreeding	1:5	rectum	17.34	1.76E+09	−	−	+	+	+
265-CU	37	no-tet	0	none	1:20	rectum	16.51	3.09E+09	−	−	−	−	−
268-CU	37	no-tet	2	rebreeding	neg	rectum	15.17	7.60E+09	−	−	+	+	+
269-CU	37	no-tet	0	rebreeding	1:20	rectum	−	−	17.69	6.08E+08	n.a	n.a	n.a
270-CU	38	no-tet	1	rebreeding	1:5	rectum	24.26	1.66E+07	18.34	3.99E+08	n.a	n.a	n.a
271-CU	38	no-tet	3	rebreeding	neg	rectum	21.47	1.09E+08	40.09	3.14E+02	n.a	n.a	n.a
273-CU	42	control	2	none	1:10	rectum	25.48	7.31E+06	−	−	+	+	+

+ Isolate carries *tetA(C)* gene/is resistant to tetracycline in vitro*,* − Isolate does not carry *tetA(C*) gene/is sensitive to tetracycline in vitro

n.a. not analyzed, CFT complement fixation test, GE genome equivalents

**Table 6:** **Characteristics of isolates from pooled fecal samples of nursery piglets (*n* = 25)**

ID	Farm ID	Study group	Location of sampling	*C. suis*		*C. pecorum*		*C. suis* susceptibility to tetracycline		
				Ct- value	DNA yield (GE/mL)	Ct-value	DNA yield (GE/mL)	*tetA(C)* gene	0.5 µg/mL	0.125 µg/mL
347-CU	1	tet	feces nursery	17.03	2.17E+09	−	−	+	+	+
192-CU	4	tet	feces nursery	17.27	1.85E+09	−	−	+	−	−
193-CU	5	tet	feces nursery	15.24	7.21E+09	−	−	−	−	−
199-CU	7	tet	feces nursery	15.12	7.85E+09	−	−	+	+	+
203-CU	9	tet	feces nursery	15.89	4.68E+09	−	−	+	+	+
206-CU	12	tet	feces nursery	14.59	1.12E+10	−	−	+	+	+
207-CU	13	tet	feces nursery	17.38	1.71E+09	−	−	+	+	+
221-CU	14	tet	feces nursery	17.51	1.57E+09	−	−	+	+	+
223-CU	15	tet	feces nursery	18.40	8.60E+08	−	−	+	+	+
228-CU	18	tet	feces nursery	19.48	4.15E+08	−	−	+	+	+
348-CU	21	no-tet	feces nursery	17.84	1.26E+09	−	−	+	+	+
302-CU	22	no-tet	feces nursery	19.57	3.92E+08	−	−	+	+	+
357-CU	23	no-tet	feces nursery	22.41	5.80E+07	−	−	+	+	+
352-CU	25	no-tet	feces nursery	19.99	2.95E+08	40.51	2.39E+02	n.a	n.a	n.a
304-CU	26	no-tet	feces nursery	18.13	1.04E+09	−	−	+	+	+
354-CU	27	no-tet	feces nursery	18.62	7.43E+08	−	−	+	+	+
356-CU	29	no-tet	feces nursery	20.42	2.22E+08	−	−	−	−	−
198-CU	32	no-tet	feces nursery	18.12	1.04E+09	−	−	+	+	+
307-CU	33	no-tet	feces nursery	19.29	4.73E+08	−	−	+	−	−
200-CU	34	no-tet	feces nursery	14.37	1.30E+10	−	−	+	+	+
201-CU	35	no-tet	feces nursery	13.08	3.09E+10	−	−	−	−	−
208-CU	37	no-tet	feces nursery	16.56	2.97E+09	−	−	+	+	+
209-CU	38	no-tet	feces nursery	15.85	4.81E+09	−	−	+	+	+
235-CU	48	control	feces nursery	22.94	4.06E+07	−	−	+	+	+
295-CU	50	control	feces nursery	20.00	2.93E+08	−	−	+	+	+

+ Isolate carries *tetA(C)* gene/is resistant to tetracycline in vitro*,* − Isolate does not carry *tetA(C*) gene/is sensitive to tetracycline in vitro

n.a. not analyzed, GE genome equivalents

For antibiotic susceptibility testing via PCR and tet screen, only pure *C. suis* isolates were included. Thus, a total of 42 isolates (one cervical, 17 rectal, 24 fecal) were evaluated for tetracycline susceptibility both by PCR and in vitro testing.

The *tetA(C)* gene, as well as phenotypic resistance, was found in isolates from all study groups. The cervical isolate carried the *tetA(C)* gene and was not susceptible to tetracycline in vitro at the tested concentrations. In total, 41 isolates from rectal swabs and nursery samples were analyzed. 85.4% (35/41) harbored the *tetA(C)* gene, 26.8% (11/41) were susceptible to tetracycline at MIC 0.125 µg/mL, one isolate displayed intermediate susceptibility, and 70.7% (29/41) were resistant in vitro.

Among 15 isolates from the tet group, 93% (14/15) carried *tetA(C)*, 80% (12/15) were resistant in vitro, one isolate showed intermediate susceptibility, and 13.3% (2/15) were susceptible at MIC 0.125 µg/mL. Of the 23 isolates from the no-tet group, 78.3% (18/23) carried *tetA(C)*, 60.8% were resistant in vitro and 39.1% (9/23) were susceptible at MIC 0.125 µg/mL. All three isolates from the control group harbored the *tetA(C)* gene and were resistant in vitro (Figure 6).

### Post-slaughter investigations of genital tracts from sows with fertility problems

**Figure 7:**
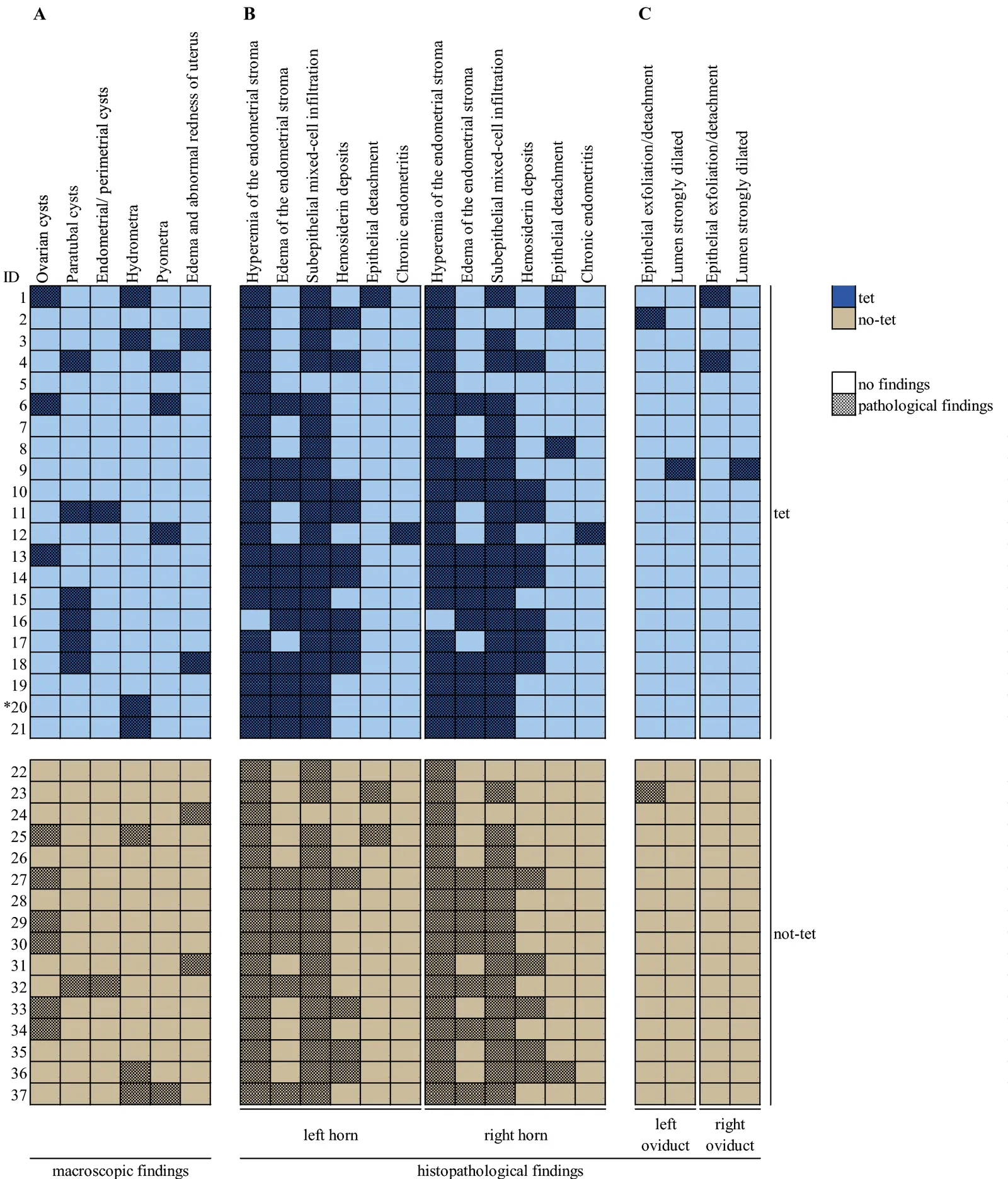
**Morphological and histopathological assessment of the porcine reproductive tract.** **A** Overview of macroscopic findings. **B** Histopathological findings of the left and right uterine horns. **C** Histopathological findings of the left and right oviducts. Specific pathological alterations and lesions are indicated by hatching. *in the corresponding Oviduct of uterus 20 *C. suis* DNA load of 1×10^1^ genome equivalents (GE)/mL DNA was detected.

**Figure 8:**
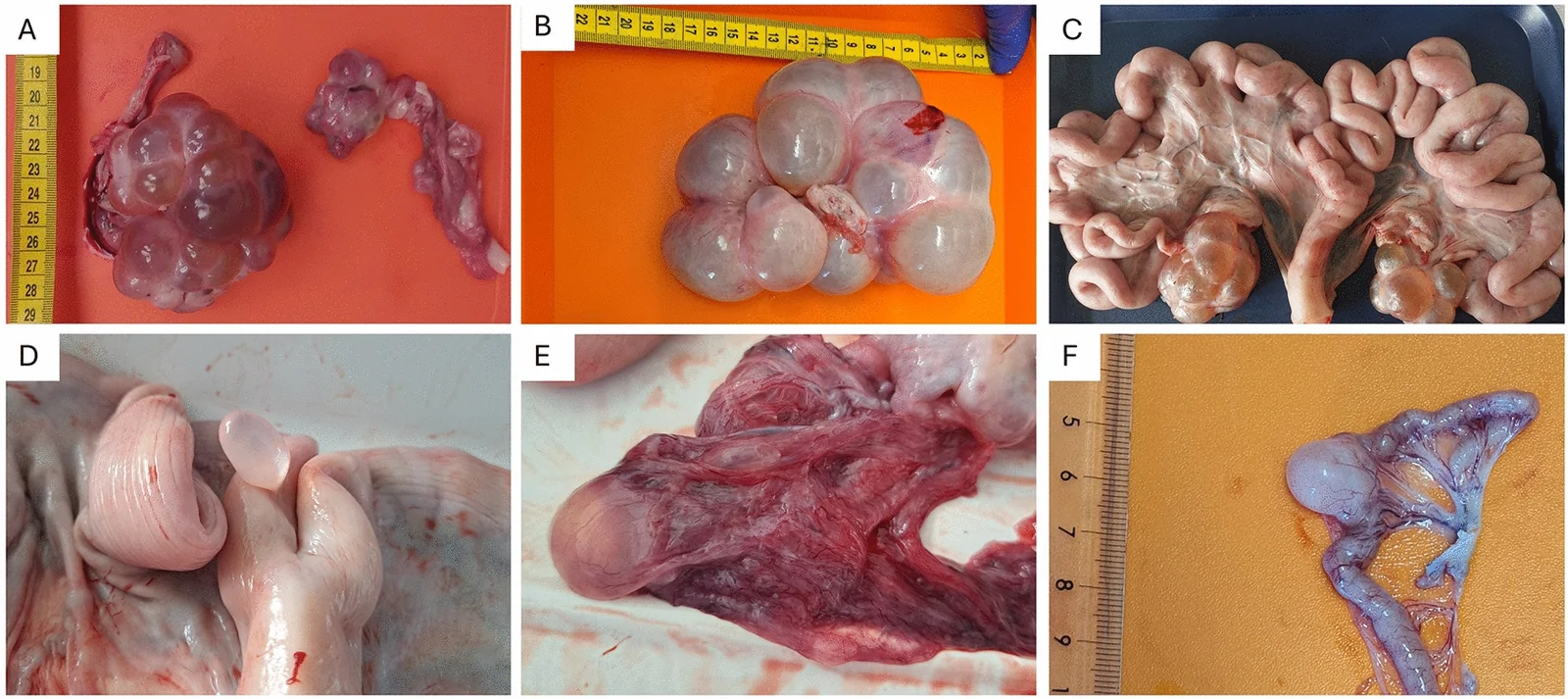
**Representative macroscopic findings of the porcine reproductive tract of slaughtered sows with fertility problems:** **A** (ID33), **B** (ID27) and **C** (ID27): Multicystic ovarian degeneration. In **C**, bilateral cystic involvement is shown in relation to the uterine horns. **D** (ID32): Cyst growing on perimetrium of left uterine horn. **E** (ID32) and **F** (ID15): Paratubal cysts.

With a single exception, all uterine and oviductal samples tested negative for *Chlamydiaceae* and subsequently *C. suis* DNA by qPCR. A low chlamydial DNA load of 1 × 10^1^ genome equivalents (GE)/mL was detected in the left oviduct of one sow (reproductive tract 20), whereas the contralateral right oviduct remained negative. Macroscopic examination of the reproductive tracts revealed several abnormalities, the most prevalent being ovarian cysts (24%) and paratubal cysts (19%). Histopathological lesions were largely consistent with physiological changes associated with the estrous cycle, including cycle-dependent inflammation, epithelial alterations, and vascular reactions. In the uterine horns, the most common findings were endometrial stroma hyperemia (97%), oedema (46–49%), and subepithelial mixed-cell infiltration (89–95%). Findings in oviducts included epithelial detachment and pronounced luminal dilation (Figure 7A–C; Figure 8; Additional file 4).

### Abortions

Six abortions of sows from five different farms (tet *n* = 2; no-tet *n* = 3) were negative for *Chlamydiaceae* DNA as determined by qPCR.

## Discussion

Genital chlamydiosis is widely considered a relevant differential diagnosis in herds experiencing reproductive disorders [40], a perception supported by recent survey data among specialized swine practitioners [16]. However, our findings that genital shedding of *C. suis* is rare, while tetracycline resistance rates are high, necessitate a critical reassessment of the clinical impact of *C. suis* on reproductive outcomes.

However, our findings of genital *C. suis* shedding being rare but tetracycline resistance rates being high, necessitate a critical reassessment of the clinical impact of *C. suis* on reproductive outcome. Much of the supporting evidence remains fragmented, relying on decades-old literature and anecdotal reports [9–15]. Crucially, to date, Koch’s postulates for *C. suis* remain unfulfilled in the context of reproductive disorders. As experimental infection studies in pregnant sows are lacking and genital infection studies have failed to induce consistent clinical reproductive symptoms under controlled conditions [41].

This uncertainty is further compounded by substantial diagnostic challenges associated with the interpretation of chlamydial detection in pigs. A major diagnostic challenge is the high environmental burden of C. suis, which increases the risk of false-positive results due to potential sample contamination. Without stringent, aseptic sampling, positive results may reflect fecal or environmental contamination rather than true infection. Furthermore, the interpretation of the clinical relevance of qPCR results can be challenging, as relatively high Ct-values are frequently observed, as demonstrated in our dataset (Additional file 1). Although cut-off values for considering samples as positive are generally defined at Ct-values below 36–38 [2], the detection of bacterial DNA does not necessarily indicate the presence of viable organisms. In our study, viable isolates were successfully recovered only from samples with Ct-values < 34 (Additional file 3), suggesting that a Ct-value of approximately 34 may represent a potential threshold for clinical relevance.

*C. abortus* and *C. psittaci* were not specifically targeted in this study, as the diagnostic approach focused on the predominant porcine species *C. suis* and *C. pecorum* [2] following *Chlamydiaceae* family-level detection. While *C. abortus* and *C. psittaci* would have been detected at the family qPCR level, the data suggest that its occurrence as a single infection in the study population is highly unlikely, although mixed infections cannot be completely excluded.

Based on this knowledge that, the pigs intestine serves as a reservoir for *C. suis *[2, 7], rectal swabs from sows–where we found relatively high rates of shedding among all study groups–were utilized as an individual-level positive control, pooled fecal samples from nursery piglets were strategically included as a herd-level positive control. This approach allowed us to confirm farm-wide pathogen circulation, regardless of the transient or intermittent shedding status of individual sows. By integrating nursery feces data, the presence of *C. suis* was successfully verified on all 50 participating farms, even when shedding of individual sows was not detected at the time of sampling. In the nursery, *C. suis* was identified in all but two farms, although shedding patterns exhibited significant variation. This may be attributable to prior antimicrobial interventions [2] or the characteristic intermittent nature of chlamydial shedding [42, 43]. Despite the dilution effect of pooling, DNA yields from nursery piglets were consistently high, indicating substantial shedding among weaners, as previously reported [2, 8]. These elevated shedding rates likely result from high infection pressure facilitated by dense stocking conditions [44]. In contrast, sows are typically maintained in less crowded or partially individualized housing, which may reduce the risk of frequent reinfection. This environmental difference, combined with the intermittent nature of chlamydial shedding [43], may further explain the limited detection of cervical shedding observed in this study.

In accordance with the findings of Unterweger et al. [21], detection of *C. suis* DNA in environmental dust of all 50 farms was moderate, and none of the positive samples yielded viable bacteria in cell culture. Showcasing that *C. suis* can be easily detected not only in feces but also in the environment of pigs, positive PCR results should be interpreted with caution.

From all study sows, cervical swabs were analyzed to reassess the role of *C. suis* in herds with reproductive issues. However, cervical chlamydial shedding was infrequent across all study groups, with DNA loads generally remaining at low levels and without association with clinical signs. The use of vaginal swabs was intentionally avoided in the present study, despite their application in previous surveys [19], as they offer limited diagnostic value due to the substantial risk of fecal and environmental contamination [45]. Given that sows are in constant contact with feces during recumbency, the detection of *C. suis* in the vaginal vestibule does not necessarily signify a true infection of the reproductive tract. Instead, it may reflect passive colonization or transient presence due to the close anatomical proximity to the anus. While an ascending route of infection remains a theoretical possibility, the mere detection of DNA in the lower urogenital tract is insufficient to establish a causal link to reproductive disorders. To mitigate these confounding factors and ensure a more representative assessment of the internal reproductive environment, cervical swabs were collected with special care using a speculum to facilitate aseptic sampling [10, 45].

To further investigate Chlamydia infections of the genital tract, farmers from both tet and no-tet groups were invited to submit genital tracts from slaughtered sows with a history of reproductive failure, alongside abortion material. Out of 37 received reproductive tracts, *C. suis* DNA was detected in only one single oviduct from a sow of tet group, without associated macroscopical or histopathological lesions. These findings contrast with a 2006 German study [10] that reported *Chlamydiaceae* DNA in 61.9% of uteri from repeat breeder sows. Such discrepancies are likely attributable to differences in PCR targets and sensitivities. Nevertheless, our results align with more recent evidence suggesting a limited role of *Chlamydia* spp. in the pathogenesis of porcine endometritis [46]. Although direct chlamydial detection within the upper genital tract was minimal, the pathological examination of slaughtered sows’ genital tracts provided valuable insights into alternative fertility-relevant pathologies. A significant proportion of the reproductive tracts exhibited alternative pathologies, including (multiple) ovarian and paratubal (Morgagni) cysts or pyometra, which could independently explain the reproductive failure in these herds. The lack of finding chlamydial DNA in abortions from farms with potential chlamydiosis contradicts earlier reports that identified Chlamydiae via immunohistochemistry [14] or Stamp staining [15]. Assuming that sufficient diagnostic material and the appropriate target tissue was analyzed—aspects that have never been investigated in animal trials—our results lead to a clear conclusion that *C. suis* was not the causative agent of abortion in the submitted cases. Our data highlight that the detection of *C. suis* DNA in rectal swabs or the presence of specific antibodies in the herd should not be misinterpreted as being linked to chlamydial-induced abortion.

As stated above, *C. suis* DNA was identified on all farms, irrespective of tetracycline treatment or the presence and severity of reproductive issues, and serological analyses revealed comparable antibody detection rates across all herds. Together, these findings indicate that, although veterinarians frequently consider *C. suis* a relevant differential diagnosis, its mere presence does not sufficiently explain the observed reproductive disorders in these herds, highlighting the importance of considering additional contributing factors and pathologies. This discrepancy necessitated a more detailed examination of the upper genital tract and abortion material. One potential explanation for the lack of differences between treated and untreated herds is the limited uterine and oviductal tissue penetration of tetracyclines administered during the breeding period, potentially facilitating local *C. suis* persistence despite treatment. Pharmacokinetic data from ruminants indicate that systemic oxytetracycline often fails to reach uterine concentrations above the minimum inhibitory concentration (MIC) for relevant pathogens, and oral bioavailability varies across compounds and species, further limiting effective drug exposure [47, 48]. These limitations may explain the lack of differences in genital detection between tetracycline-treated and untreated herds in this study.

The diagnostic value of serology in this context appears equally limited. While some former studies reported high antibody detection rates irrespective of clinical status [9], others found associations with reproductive outcomes [12, 49]. More recent data using CFT demonstrated very low seroprevalence [19]. These discrepancies are likely attributable to methodological differences, including choice of serological assays. In particular, ELISA and CFT target the chlamydial lipopolysaccharide (LPS) and are prone to cross-reactivity between species [50]. Furthermore, the lack of validated CFT cut-off values complicates clinical interpretation. In the present study, antibody titers were generally low, potentially reflecting non-specific binding or cross-reactions [50] rather than active infection. Importantly, no association was observed between antibody detection and reproductive performance, indicating that serological findings—particularly those obtained by CFT—are of limited value for assessing the clinical relevance of *C. suis* in reproductive disorders. Nonetheless, we found antibody titers of 1:40 and higher only in sows from problem farms, and not in the control group. To date, it remains unclear whether antibodies measured by IFA, ELISA, or CFT confer protective immunity, although micro-immunofluorescence (MIF) titers may correlate with neutralizing antibodies [32].

We identified both seropositive and seronegative animals among sows with cervical shedding, also no association was found between rectal shedding and antibody status. Notably, parity had a negative effect on expression of antibodies, suggesting early exposure to *C. suis, w*hich is corroborated 96% pathogen prevalence in nurseries, with piglets colonized as early as four to five weeks of age. Parity was also found to have a negative effect on rectal shedding, suggesting that older sows may develop an age-dependent decreased susceptibility to reinfection, consistent with observations in human *C. trachomatis* infections [51, 52]. Furthermore, the observed negative association between parity and reproductive disorders likely reflects a survival bias rather than age-related chlamydial immunity. Sows with recurrent reproductive failure are typically culled early [27, 53, 54], leaving older parity groups as a 'selected' population of fertile animals. Additionally, confounding factors such as the "second litter syndrome" [55] must be considered, as they predispose younger sows to reproductive challenges independent of their chlamydial status.

In this study special attention was given to *C. suis* and its unique capacity to acquire tetracycline resistance [1, 23], a trait that potentially influences both pathogen persistence and therapeutic outcomes. In Austrian swine production, tetracyclines remain among the most frequently used antibiotic classes. National sales data underscore this, with nearly 10 tons dispensed for porcine use in 2023 [56]. In cases of suspected genital chlamydiosis tetracyclines are often administered around the breeding period in herds experiencing reproductive disorders [16, 27]. To evaluate whether such antimicrobial exposure influences *C. suis* prevalence, clinical associations, shedding patterns, or in vitro resistance, herds were stratified based on their routine tetracycline usage. Additionally, *C. pecorum* was included as a secondary target due to its documented occasional presence in previous surveys [2].

Although no association between study group and shedding was identified, a numerical difference regarding the isolation process of *C. suis* was observed between study groups, with nearly twice as many successfully isolated strains from the no-tet group (*n* = 29) compared to the tet group (*n*=17). While this lower frequency of isolation success in the tet group might superficially suggest an effect of routine antimicrobial prophylaxis, such data must be interpreted with caution considering the isolation process. Crucially, the only viable cervical *C. suis* isolate recovered in this study originated from a sow within the tet group, marking, to our knowledge, the first reported description of a porcine genital isolate. This finding is of high clinical significance, as it demonstrates that routine antimicrobial use not only fails to eliminate *C. suis* from the herd but, more critically, may actively promote the selection and persistence of resistant strains [25] within the reproductive tract. Subsequently we found phenotypic and genotypic tetracycline resistant strains in all study groups. The potential risk of false-positive PCR results, resulting from the detection of the *tetA(*C) gene, which is not exclusively restricted to *C. suis* and may also occur in other Gammaproteobacteria [57], was minimized by including only visually uncontaminated *C. suis* isolates. The discovery of the resistant genital isolate is mirrored by high rates of tetracycline resistance across all study groups, with *tetA(C)* prevalence being higher on tet farms (93.3%, 14/15) compared to no-tet farms (78%, 18/23). Phenotypic resistance was correspondingly also observed more frequently in tet farms (80% vs. 60%).

Contrasting Seth-Smith et al. [58] who reported only phenotypically resistant *tetA(C)*-positive isolates, we found some *tetA(C)*-positive isolates, which remained susceptible in vitro. This indicatrices a complex coexistence of resistant and sensitive strains—potentially due to fitness advantages of the latter [59]—and confirms that the presence of *tetA(C)* alone does not necessarily correlate with phenotypic tetracycline resistance, as previously reported by Wanninger et al. [25] and Zubler et al. [8].

The occurrence of both susceptible and resistant strains within single herds underscores the complexity of clinical management, particularly given the lack of standardized treatment guidelines and the high variability in field protocols regarding dose and duration [27]. Furthermore, with the potential for tetracycline resistance in other chlamydial species remaining unexplored [23], our results highlight the urgent need for comparative genomic analyses to determine if genital isolates exhibit site-specific adaptations and for the development of evidence-based susceptibility screening and treatment protocols.

## Conclusion

In farms with suspected genital chlamydiosis, genital shedding of *C. suis* was minimal, despite the successful recovery of the first viable porcine genital isolate in cell culture. While rectal shedding in sows was common, and detection in nursery piglet feces was consistently high, with no farm found free of *C. suis,* neither shedding patterns nor antibody prevalence were associated with clinical reproductive status or antimicrobial treatment groups. Notably, parity negatively influenced rectal shedding, antibody expression, and clinical signs, suggesting age-related exposure dynamics. High tetracycline resistance was observed across all study groups, with both susceptible and resistant strains coexisting within the same herd. The use of tetracyclines may therefore fail to achieve the intended therapeutic effect in cases of genital chlamydiosis and may additionally promote the selection of resistant strains. Pathological examination of reproductive tracts from slaughtered sows rarely identified *C. suis* DNA; however, it proved valuable for diagnosing alternative pathologies, such as ovarian or paratubal cysts, which likely accounted for the observed reproductive failure. Consequently, for the diagnosis of acute genital chlamydiosis, species-specific qPCR of cervical swabs remains the recommended approach, while serology is limited to assessing prior exposure rather than active clinical disease.

## Supplementary Information


 **Additional file 1 Overview PCR results cervix and rectum swabs from sows.** **Additional file 2 Overview PCR results dust samples and nursery feces samples.****Additional file 3. Overview PCR and antimicrobial susceptibility results of** ***Chlamydiaceae/C. suis*** **isolates.** **Additional file 4. PCR results and macroscopic and histopathological findings in reproductive tracts from slaughtered sows with fertility problems.**

## Data Availability

The datasets used and/or analyzed during the current study are available from the corresponding author on reasonable request.
