# Supplementary material for: Influence of Chlamydia suis on reproductive outcome in farms with and without tetracycline treatment of sows
Source: Vet Res. 2026 Aug 31;57:162. doi: 10.1186/s13567-026-01831-w (PMC13527907; doi:10.1186/s13567-026-01831-w)
Supplement: Supplementary file 1 [file 13567_2026_1831_MOESM4_ESM.pdf]

Additional file 4: PCR results, macroscopic and histopathological findings in reproductive tracts from slaughtered sows with fertility problems

	findings in total no. of uteri (n=37)		findings in uteri from tet farms (n = 21)		findings in uteri from no-tet farms (n = 16)	
	no. of lesions found	(%)	no. of lesions found	(%)	no. of lesions found	(%)
**Macroscopic findings**						
total			21		16	
Ovarian cysts	9	(24)	3	(14)	6	(38)
Paratubal cysts	7	(19)	6	(29)	1	(6)
Endometrial/perimetrial cysts	2	(3)	1	(5)	1	(0)
Hydrometra	7	(19)	4	(19)	3	(19)
Pyometra	4	(11)	3	(14)	1	(6)
Edema and abnormal redness of uterus	4	(11)	2	(10)	2	(13)
**Histopathological findings**						
**Uterus horn left side**						
Hyperemia of the endometrial stroma	36	(97)	20	(95)	16	(100)
Edema of the endometrial stroma	17	(46)	11	(52)	6	(38)
Subepithelial mixed-cell infiltration	35	(95)	20	(95)	15	(94)
Hemosiderin deposits	13	(35)	9	(43)	4	(25)
Epithelial detachment	3	(8)	1	(5)	2	(13)
Chronic endometritis	1	(3)	1	(5)	0	(0)
**Uterus horn right side**						
Hyperemia of the endometrial stroma	36	(97)	20	(95)	16	(100)
Edema of the endometrial stroma	18	(49)	11	(52)	7	(44)
Subepithelial mixed-cell infiltration	33	(89)	19	(90)	14	(88)
Hemosiderin deposits	13	(35)	8	(38)	5	(31)
Epithelial detachment	4	(11)	3	(14)	1	(6)
Chronic endometritis	1	(3)	1	(5)	0	(0)
**Oviduct left side**						
Epithelial exfoliation/detachment	2	(5)	1	(5)	1	(6)
Lumen strongly dilated	1	(3)	1	(5)	0	(0)
**Oviduct right side**						
Epithelial exfoliation/detachment	2	(5)	2	(10)	0	(0)
Lumen strongly dilated	1	(3)	1	(5)	0	(0)
**Positive PCR results**						
Uterus horn left side	0	(0)	0	(0)	0	(0)
Uterus horn right side	0	(0)	0	(0)	0	(0)
Oviduct left side	1	(3)	1	(5)	0	(0)
Oviduct right side	0	(0)	0	(0)	0	(0)
